# The Impact of Sociodemographic Factors on Job Satisfaction and Professional Burnout among Nurses in Urology Departments

**DOI:** 10.3390/nursrep14020068

**Published:** 2024-04-10

**Authors:** Katarzyna Jarosz, Agnieszka Młynarska

**Affiliations:** Department of Gerontology and Geriatric Nursing, School of Health Sciences, Medical University of Silesia, 40-55 Katowice, Poland; amlynarska@sum.edu.pl

**Keywords:** job satisfaction, professional burnout, sociodemographic factors, nurse, urology

## Abstract

(1) Background: Job satisfaction and professional burnout directly impact human life, depending on various professional, non-professional, and private determinants. Nurses, in particular, are highly susceptible to experiencing professional burnout, which, when combined with job satisfaction, significantly affects the quality of their services. This study aimed to assess the level of job satisfaction and job burnout among nurses working in urology departments, as well as the impact of sociodemographic factors. (2) Methods: The study involved 130 nurses working in urology departments in Poland. Researchers conducted an anonymous questionnaire comprising a sociodemographic section and two standardized questionnaires: the Link Burnout Questionnaire (LBQ) and the Scale of Job Satisfaction (SSP). (3) Results: The study group demonstrated an average level of job satisfaction (17.23 points) and an average level of professional burnout, indicating potential symptoms of professional burnout such as psychophysical exhaustion (22.29 points), lack of commitment to patient relationships (20.02 points), feelings of professional ineffectiveness (17.37 points), and disappointment (19.66 points). (4) Conclusions: The levels of job satisfaction and professional burnout among nurses in urology departments are comparable to those in other departments and countries. Medical facilities should take into account factors influencing job satisfaction and the risk of professional burnout when addressing employment conditions.

## 1. Introduction

Employment holds a crucial position in an individual’s life, not solely from a financial standpoint but also in terms of its impact on social and psychological well-being. Job satisfaction directly influences one’s life, self-esteem, and sense of fulfillment, playing an essential role in the ability to achieve and maintain a respected status in society, while also nurturing self-realization, personal growth, and interpersonal connections [1]. Nurses represent a distinct professional group that is especially susceptible to experiencing occupational burnout. This vulnerability primarily arises from the intrinsic nature of their profession, involving the provision of care to the ill under demanding work conditions that necessitate perpetual physical and mental readiness. Nurses routinely confront emotionally demanding situations such as patient loss, the risk of patient suffering, and a profound responsibility for others’ well-being. However, notwithstanding these challenges, nurses diligently fulfill their duties with professionalism and main high standards of care [2]. Nurses possessing the requisite qualifications and actively engaging in their profession are recognized as proficient staff members endowed with specific competencies. They are empowered to make autonomous decisions and assume responsibility for coordinating patient care [3]. Urological nurses need to have wide, specialized knowledge and skills such as active listening and individualizing patient care. At each stage of care, a nurse serves as an educator. Urological nurses assist patients, particularly men, in coping with the negative effects of their disease, such as low testosterone levels, decreased libido, sexual disorders, or difficulty with urination—issues that can be quite embarrassing for patients. Nurses are responsible for preparing patients and their families for home care by providing education and emotional support [4].

In social occupations involving continual interpersonal contact with numerous individuals, professional burnout is frequently experienced [5]. According to Maslach and Jackson, professional burnout is commonly observed in professions such as nursing, teaching, medicine, law enforcement, and social work [6]. Olley’s research additionally corroborates the vulnerability of nurses to burnout [7]. The study conducted by Bashirian et al. revealed that nurses experienced burnout more frequently than other health workers following the COVID-19 pandemic [8]. Nurses play a crucial role in healthcare systems’ response to this pandemic, as they are frontline workers directly engaged in patient treatment and care. The COVID-19 pandemic has led to increased burnout among nurses, which has implications for patient safety and healthcare-provider productivity. Previous studies have suggested that this burnout may result from physical exhaustion due to excessive workload, shortages of medical staff and necessary equipment for COVID-19 patient care, increased patient mortality rates, inequity, lack of mutual respect, differing organizational values, insufficient organizational support, extended shifts working closely with infected patients, and fear of infection or transmission [9].

Professional burnout is a global issue and has been officially recognized as a professional phenomenon in the International Classification of Diseases (ICD-11) with the assigned code QD85 [10]. Research studies conducted worldwide demonstrate that professional burnout significantly affects numerous nurses. In Spain, for instance, 39.8% of nurses experience professional burnout [11], while in the USA the prevalence is even higher, at 54.1% [12]. International research focusing on surgical departments has revealed varying rates of professional burnout across different countries, including England (42%), Finland (22%), Belgium (25%), Germany (30%), Poland (40%), Ireland (41%), Norway (24%), Spain (29%), the Netherlands (10%), and Sweden (15%) [13]. A study by Shah et al. indicated that professional burnout was the primary reason behind quitting for 31.5% of nurses quit and the primary reason for 43.4% of nurses who contemplated leaving their positions. Nurses who resigned from hospital employment were twice as likely to experience burnout compared to those working in other types of medical facilities. Among nurses who considered quitting their jobs, the likelihood of experiencing burnout was 80% [14].

In the 1970s, Freudenberger initially described professional burnout as an exhaustion resulting from excessive demands [15]. Maslach and Jackson subsequently developed the most widely used and popular definition of professional burnout, characterizing it as a syndrome comprising emotional exhaustion, depersonalization, and reduced personal accomplishment. This syndrome commonly affects individuals occupying specific roles involving various interactions with others [16]. The progression of professional burnout is gradual, representing the final stage in a continuum that commences with engagement and motivation. Its onset is challenging to determine [17]. It is often observed that burnout becomes apparent when employees experience a lack of job satisfaction [18]. As the burnout process develops, individuals may lose their sense of purpose and become disillusioned, ultimately leading to feelings of disappointment [17]. Chronic or persistent stress serves as a foundational factor in the development of burnout. Burnout can be viewed as the body’s response to ongoing stress [18]. According to Selye, stress is a condition that triggers a non-specific physiological response in the body to external demands. Initially, there is a mobilization phase as the body attempts to adapt and restore balance. However, if stress persists for an extended period, a general adaptation syndrome occurs, characterized by three phases: alarm, resistance, and exhaustion. Ultimately, this process results in a lack of energy and an inability to meet the demands placed on the individual [19].

Burnout is often a hidden struggle or a concealed challenge because people may not recognize it or may not want to acknowledge that they are experiencing it. They might not feel comfortable speaking up about their problem. Burnout can gradually accumulate over time as individuals face chronic stress, high work demands, long hours, a lack of work–life balance, and insufficient support in the work environment. Those who experience burnout may feel embarrassed to admit it, often believing that it is a sign of weakness or a personal failure. Additionally, they may face stigma in the workplace due to their illness, which discourages them from seeking help or opening up to others [20].

Burnout factors can be categorized into several groups, including organizational factors, personal factors, stress coping styles, sociodemographic factors, and lifestyle [21]. Professional burnout generally pertains to the relationship between an employee and their work. The primary syndromes associated with professional burnout, specifically manifesting in connection to work, include feelings of tension, hypersensitivity, and overactivity, accompanied by psychophysical exhaustion [22]. Gender, marital status, and age are among the main factors influencing occupational burnout [23]. Psychoemotional factors such as stress, anxiety, and depression are also significant contributors [24]. Additionally, low income, shift work, overwork [25], performing tasks that exceed an employee’s skills, and engaging in repetitive and monotonous work can contribute to burnout [18]. The presence of occupational burnout and the associated mental and somatic symptoms can be influenced by a situation in which nurses are engaged and ambitious in their work but do not receive appropriate compensation [26]. Education is connected to various aspects of work, including higher prestige, social status, the responsibility and stressfulness of the job, increased requirements for employees, and greater demands in the work environment [27].

In the field of urology, the demanding nature of surgical practice, high patient volume, and administrative burdens are additional factors contributing to burnout. Burnout can also manifest itself in physical symptoms, such as headaches, stomachaches, and exhaustion, which can indicate other causes, making burnout difficult to diagnose. Healthcare providers may hesitate to seek help or speak up about their struggles because they fear being perceived as weak or inadequate if they admit to experiencing burnout. In some workplaces, healthcare workers are expected to be constantly available and work overtime, leading to burnout becoming normalized and ignored. Some employers may view burnout as an individual problem rather than a systemic issue, which hinders effective efforts to recognize and eliminate root causes in the workplace. Organizational factors also impact the occurrence of burnout. Healthcare organizations often prioritize productivity and efficiency, resulting in even greater demands on healthcare workers. Administrative pressure, lack of resources, long working hours, and high patient loads may contribute to burnout. However, the significance of burnout may be overlooked or ignored due to financial limitations or organizational priorities. The limited awareness and understanding of burnout can lead to it being misunderstood or underestimated in terms of its impact on healthcare providers and patient care [20].

Regardless of whether a country is developing or developed, researchers have long focused on health providers’ job satisfaction. Job satisfaction has been studied more than any other variable in healthcare because of its influence on individual healthcare professionals and the organization. Despite nurses being a significant part of the healthcare system and being involved in approximately 90% of direct patient care activities, there are significant gaps in research related to nurses. Although researches have been more prevalent over the last 80 years, there is still much to explore in understanding nurses’ experience and there is no consensus on the definition of job satisfaction [28].

In the 1930s, the first publications about job satisfaction were released. This topic has been extensively studied for many years [29]. In the 1980s, job satisfaction underwent renewed research, particularly regarding its correlation with other behaviors in the workplace and other spheres of employees’ lives [30]. There are numerous definitions of job satisfaction. One of them, formulated by Zalewska, asserts that the level of job satisfaction is determined by an employee’s assessment of the work and their relationship to it [31]. According to Shultz et al., job satisfaction is characterized as a subjective evaluation of positive and negative feelings and attitudes connected to an employee’s duties. Positive job satisfaction occurs when an employee is content with their duties and working conditions, while negative job satisfaction refers to the opposite [32].

There are three primary groups of factors that influence job satisfaction: individual factors related to the employee, occupational factors, and non-occupational factors [33]. Non-occupational factors encompass general life satisfaction, family, health, place of residence, social support, and financial status [34]. Factors such as staff shortages, excessive patient workload per nurse, working environment, overtime work, shift work, overwork, and lack of relationships within the therapeutic team can negatively impact job satisfaction [35]. In the literature, the principal factors identified as affecting job satisfaction include physical health, satisfaction with salary, overwork, quality of life, seniority, staffing levels during shifts, satisfaction with family, satisfaction with relationships among staff, satisfaction with cooperation with a supervisor and their actions, and satisfaction with working facilities [36].

A low level of job satisfaction causes a low quality of nurses’ services and a lack of engagement in their duties [37]. Employees who are characterized by a high level of job satisfaction are more engaged in their duties, strive to achieve the company’s goals, and exhibit greater creativity [38]. Research has shown a correlation between the perceived level of job satisfaction and patient satisfaction with nursing care [39].

Job satisfaction and professional burnout have been extensively researched for many years across various fields and occupational groups. Research on these phenomena is crucial, particularly given the evolving situation of nurses’ employment. In Poland, there has been a continuous decrease in the number of actively practicing nurses, while the average age of nurses continues to rise. Many individuals complete their nursing studies but do not pursue work in the nursing profession, or they choose to move abroad. According to projections by the Main Chamber of Nurses and Midwives in Poland, the number of nurses is expected to decrease to 3.81 nurses per 1000 citizens by 2030, compared to 5.04 nurses per 1000 citizens in 2020. Approximately 50% of actively practicing nurses work in hospitals [40]. Buchan and Aiken proposed a theory related to staff shortages, suggesting that the issue lies not in a lack of nursing graduates but rather in a shortage of nurses willing to work under current conditions [41]. Studying the factors that impact the growth of professional burnout and the decrease in job satisfaction can lead to valuable changes that encourage people to pursue nursing studies and create appropriate conditions for providing high-quality services and ensuring patient safety.

Studies of job satisfaction and professional burnout have been conducted among nurses working in various departments, including intensive care units and internal departments. However, urology departments are often omitted or combined with surgical departments, which is why we have chosen to focus on urology departments. These departments offer a unique perspective for studying job satisfaction and burnout in nursing research. Urological nurses possess specific competences and frequently work in diverse settings, such as outpatient facilities, hospital wards, and rehabilitative facilities. They cover ultra-specialized fields like uro-oncology, urogynecology, pediatric urology, sexual disorders, urinary incontinence, and urostomy rehabilitation. In these areas, nurses require specific skills and competences to perform occupational tasks. They assist doctors during medical procedures, prepare patients for examinations and surgeries, conduct procedures like catheterization, and educate patients on topics such as urostomy care, wound dressing changes, proper diet, and exercise. Beyond practical skills, nurses also serve as psychological support for patients, particularly in cases of oncological diseases or sexual disorders. Additionally, nurses are obligated to continually update their knowledge and enhance their competences [42]. Urological nurses treat a wide range of conditions, from bladder cancer to chronic bladder incontinence, and they often deal with patients’ embarrassment and discomfort related to bladder or prostate issues. Sympathy and understanding are integral parts of their skill set. When interacting with new patients, urological nurses draw upon their extensive knowledge and patient interactions to provide reassurance and help individuals cope [43].

This high level of specialization significantly impacts the perception of the occupation and wage considerations, satisfaction, and burnout. Additionally, working as a urology nurse is mentally taxing due to the constant contact with oncological patients and their intimate sphere, particularly related to sexuality. Another factor influencing job satisfaction and burnout was the COVID-19 pandemic, which required medical staff to work under increased pressure and in uncomfortable conditions. Medical staff worked overtime, hospital wards were overcrowded, and staff witnessed their patients’ deaths more frequently than before [44].

Therefore, the aim of this study was to assess the influence of sociodemographic factors (place of residence, forms of postgraduate education, work pattern, number of patients per nurse during a shift, financial satisfaction, number of diseases faced by a nurse) on professional burnout and job satisfaction among nurses working in urology departments. 

The main research hypotheses are as follows: Sociodemographic factors significantly impact job satisfaction and professional burnout; 1.1. Increased financial satisfaction and a higher level of postgraduate education lead to higher job satisfaction and reduced professional burnout; 1.2. Conversely, an increase in the number of patients per nurse, the number of diseases faced by a nurse, and specific work patterns contribute to higher professional burnout and decreased job satisfaction.

## 2. Materials and Methods

### 2.1. Study Design

The conducted study was a cross-sectional investigation comprising a research group of 130 nurses employed in urology departments across Poland. The report of the Supreme Council of Nurses and Midwives in 2021 indicates that there were approximately 239,387 nurses in Poland, equating to an average of 5.2 people per nurse. Public sources do not specify the number of nurses working in urology departments. According to the Central Statistical Office, there were 3177 patient beds in urology departments in 2021. Additionally, as of 1 January 2019, there should be 0.7 full-time nurses employed per patient bed in departments in which surgeries are performed. To provide context, other departments had the following bed counts: 34,576 in surgical departments and 8571 in cardiological departments. It is important to note that Poland faces a constant shortage of nurses, leading many to work more than full-time hours and take on overtime to ensure continuity of patient care. Considering the challenges posed by the COVID-19 pandemic and the difficulty in accessing nurses, the inclusion of 130 nurses in this study holds significance [45,46,47].

### 2.2. Procedure

The data for the study were collected over a period of 3 months, from March to May, 2021. The questionnaires were provided to nurses in both paper and online formats in urology departments. The data collected from the questionnaires were then entered into an Excel spreadsheet for statistical analysis. Nurses were informed that participation in the study was voluntary and anonymous. Prior to the assessment, consent was obtained from the nurses. The study obtained prior approval from the Bioethics Committee of the Medical University of Silesia in Katowice (Ethical Number: PCN/CBN/0052/KB/32/22).

### 2.3. Data Analysis

Initially, correlations between independent variables (predictors) were examined to identify and eliminate variables that were strongly correlated with others. Subsequently, the impact of the remaining predictors on job satisfaction and professional burnout, as well as the effectiveness of the independent variables in explaining the dependent variable, were tested. The significance level was set at *p* = 0.05, with results of *p* < 0.05 indicating significant relationships between the variables.

### 2.4. Instruments

The study employed an anonymous questionnaire comprising three components: a metric section, the standardized Link Burnout Questionnaire (LBQ), and the Scale of Job Satisfaction (SSP). Both questionnaires were utilized in the Polish language version.

The Scale of Job Satisfaction (SSP) was adapted from the Scale of Life Satisfaction (SWLS) developed by Diener et al. in 1985. The SWLS allows researchers to assess the cognitive aspect of general life satisfaction. The Scale of Job Satisfaction was developed in Poland by Zalewska in 2003 and is characterized by its high convergent and differential validity. It also demonstrates high internal consistency (Cronbach’s alpha above 0.80). The life sphere statements in the SWLS questionnaire were modified into work-related statements. The job satisfaction study is based on five statements about work, each of which is assessed on a 7-point scale, as follows: 1—I strongly disagree, 2—I don’t agree, 3—I rather disagree, 4—It’s hard to say whether I agree or disagree, 5—I rather agree, 6—I agree, 7—I definitely agree. Participants can score between 5 and 35 points, with higher scores indicating a higher level of job satisfaction [30].

The Link Burnout Questionnaire (LBQ) was developed in Italy by Massimo Santinello in 2008. The Polish version of this questionnaire was prepared by Jaworowska in 2014. This tool is specifically designed for assessing professional burnout among individuals involved in teaching and helping professions. The Polish version is compatible with the original version and is characterized by high reliability. Cronbach’s alpha values for the individual parts of the questionnaire are as follows: 0.77 for psychophysical exhaustion, 0.69 for a lack of commitment to relationships with patients, 0.85 for disappointment, and 0.68 for a sense of professional ineffectiveness. The questionnaire consists of 24 statements related to work and feelings about work. A 6-point scale was used to assess professional burnout, ranging from “never” to “every day”. The LBQ is utilized to study four areas: the psychophysical area (exhaustion–energy), relationships (lack of engagement–engagement), professional competences (ineffectiveness–effectiveness), and existential expectations (disappointment–satisfaction). Participants can receive scores ranging from 6 to 36 points, which are divided into three categories: 6–10 points represent a low outcome with no symptoms of occupational burnout, 11–25 points indicate an average outcome with a possibility of experiencing professional burnout symptoms, and 26–36 points signify a high outcome with the presence of job burnout symptoms at a high level [48].

### 2.5. Participants

The study involved 130 nurses employed in urology departments in Poland. The inclusion criteria comprised consent to participate in the examination, occupation as a nurse, employment in the urology department, and a minimum work experience of 6 months, regardless of the number of hours worked (full-time/part-time). The study specifically included urology department nurses, while nurses working in operating theaters or surgical departments were excluded. The exclusion criteria included lack of consent and incomplete response to the questionnaire. 

Most of the respondents were women (98.5%), and the average age of the respondents was 37.78 years (±11.86 years). The average total length of service was 13.31 years (±12.63 years), and the average length of service in the urology department was 7.71 years (±10.29 years). The largest proportion of nurses held a bachelor’s degree (57.7%), and 46% of the respondents did not have additional postgraduate education. The majority of respondents held one job (61%), with an average of 1.45 jobs (±0.6 jobs), and they worked in a 12 h shift system (83%). On average, there were 9.56 patients per nurse in the urology department (±4.62 patients), and the nurses reported an average of 4.23 diseases (±2.46). Further details are provided in Table 1.

## 3. Results

### 3.1. Basic Descriptive Statistics

The outcomes in each domain of professional burnout fall within the range of 11–25 points, indicating an average outcome and the potential to experience symptoms of professional burnout. The scores for the specific domains are as follows: psychophysical exhaustion—22.29 points; lack of commitment to relationships with patients—20.02 points; ineffectiveness—17.37 points; disappointment—19.66 points. The overall score for job satisfaction is 17.23 points (±6.25 points), indicating average job satisfaction. Further details are presented in Table 2.

### 3.2. Correlation Analysis

A correlation analysis was conducted to determine the presence of correlations among the independent variables (predictors) and to identify variables that correlate strongly with others. Further details are presented in Table 3.

A correlation analysis using Spearman’s test revealed strong correlations between certain variables, necessitating their exclusion from the analysis. These variables included age, seniority in the nursing profession, tenure, and marital status. Furthermore, education was found to strongly correlate with the number of forms of postgraduate education. As a result, the decision was made to remove the education variable and retain the variable for postgraduate education, as the latter proved to be significant in the regression model, unlike education. Similarly, the sick leave variable exhibited a strong correlation with the number of diseases, which had a significant impact on the obtained results. Consequently, the sick leave variable was removed. Additionally, the variable for the number of jobs, which was irrelevant to further analysis, correlated strongly with the variable for the number of patients, leading to the removal of the former. Lastly, the strong correlation between the variables for satisfaction with the financial situation and willingness to change jobs necessitated the removal of the willingness to change jobs variable from the regression analysis. These exclusions were essential to maintain methodological correctness, as one of the assumptions regarding the regression model is the independence of observation errors, or the absence of a correlation.

### 3.3. Analysis of Variance

The result of the F-test is statistically significant and indicates that sociodemographic factors have a statistically significant impact on job satisfaction (F(6.123) = 7.257; *p* < 0.001), psychophysical exhaustion (F(6.123) = 6.170; *p* < 0.001), lack of commitment to relationships with patients (F(6.123) = 5.006; *p* = 0.001), feelings of ineffectiveness (F(6.123) = 2.903; *p* = 0.011), and disappointment (F(6.123) = 5.413; *p* < 0.001). The details are presented in Table 4.

### 3.4. Regression Analysis

A meticulous analysis revealed a significant impact of financial satisfaction on job satisfaction. The regression coefficient was 0.352 (t(123) = 4.380, *p* < 0.001), indicating that for every one-unit increase in financial satisfaction, job satisfaction increases by 0.352 points. Additionally, the analysis unveiled a significant impact of the work pattern on job satisfaction. The regression coefficient was −0.160 (t(123) = −2.036, *p* = 0.044), suggesting that for every one-unit increase in work pattern, job satisfaction decreases by 0.160 points. Further details can be found in Table 5.

A comprehensive analysis revealed a significant impact of the number of patients per nurse on psychophysical exhaustion. The regression coefficient was 0.180 (t(123) = 2.196, *p* = 0.030), indicating that, for each additional patient, psychophysical exhaustion increases by 0.180 points. Furthermore, the analysis demonstrated a significant influence of financial satisfaction on psychophysical exhaustion. The regression coefficient was −0.211 (t(123) = −2.575, *p* = 0.011), implying that, with every one-unit increase in financial satisfaction, psychophysical exhaustion decreases by 0.211 points. The analysis also indicated a significant impact of the number of diseases on psychophysical exhaustion, with a regression coefficient of 0.269 (t(123) = 3.315, *p* = 0.001). This suggests that, for each additional disease, psychophysical exhaustion increases by 0.269 points.

A detailed analysis revealed a significant impact of the number of patients per nurse on the lack of commitment to relationships with patients. The regression coefficient was 0.197 (t(123) = 2.353, *p* = 0.020), indicating that, for each additional patient, the lack of commitment to relationships with patients increases by 0.197 points.

The analysis also showed a significant impact of financial satisfaction on the lack of commitment to relationships with patients. The regression coefficient was −0.320 (t(123) = −3.818, *p* < 0.001). This means that, for every one-unit increase in financial satisfaction, the lack of commitment to relationships with patients decreases by 0.320 points.

A precise analysis revealed a significant impact of the work pattern on the feeling of ineffectiveness. The regression coefficient was 0.203 (t(123) = 2.375, *p* = 0.019). This means that, for each one-unit increase in work pattern compared to working on one shift, the feeling of ineffectiveness increases by 0.203 points. Additionally, the analysis revealed a significant impact of financial satisfaction on the feeling of ineffectiveness. The regression coefficient was −0.222 (t(123) = −2.536, *p* = 0.012). This indicates that, for every one-unit increase in financial satisfaction, the feeling of ineffectiveness decreases by 0.222 points.

A comprehensive analysis showed a significant impact of the number of forms of postgraduate education on disappointment. The regression coefficient was −0.189 (t(123) = −2.314, *p* = 0.022). This means that, for each one-unit increase in the number of forms of postgraduate education, the feeling of disappointment decreases by 0.189 points. Furthermore, the analysis revealed a significant impact of financial satisfaction on disappointment. The regression coefficient was −0.227 (t(123) = −2.731, *p* = 0.007). This indicates that, for every one-unit increase in financial satisfaction, the feeling of disappointment decreases by 0.227 points. Additionally, there is a significant impact of the number of diseases on disappointment. The regression coefficient was 0.217 (t(123) = 2.634, *p* = 0.010). This means that, for each additional disease, the feeling of disappointment increases by 0.217 points. Details are presented in Table 5.

### 3.5. Fit Model

The adjusted *R*^2^ value of 0.225 indicates that the model explains 22.5% of the variance in job satisfaction in the SSP. The details can be found in Table 6.

Similarly, the adjusted *R*^2^ value of 0.194 suggests that the model explains 19.4% of the variance in psychophysical exhaustion. The adjusted *R*^2^ value of 0.157 indicates that the model explains 15.7% of the variance in the lack of commitment to relationships with patients. The adjusted *R*^2^ value of 0.081 suggests that the model explains 8.1% of the variance in the feeling of ineffectiveness. Lastly, the adjusted *R*^2^ value of 0.170 indicates that the model explains 17.0% of the variance in the feeling of disappointment.

## 4. Discussion

The studied group of nurses exhibited an average level of job satisfaction and professional burnout, suggesting the possible presence of symptoms related to professional burnout. Our study revealed that financial satisfaction and work patterns significantly influenced job satisfaction. Additionally, the number of patients per nurse, financial satisfaction, and the number of diseases were found to impact psychophysical exhaustion. The number of patients and financial satisfaction were associated with a lack of commitment to patient relationships. Furthermore, work patterns and financial satisfaction influenced feelings of ineffectiveness. Additionally, the number of postgraduate education programs and financial satisfaction impacted feelings of disappointment.

This study represents the first investigation conducted among nurses specifically working in urology departments. While previous studies have examined surgical departments, which may encompass urology departments due to their specialized nature, our analysis includes novel factors, such as the place of residence, number of postgraduate education programs, and number of diseases treated by nurses. Conversely, factors like work patterns and financial satisfaction have been previously explored in other studies.

In Medscape’s 2020 National Physician Burnout and Suicide Report, urology was once again found at the top of the list, with a 54% prevalence of burnout. In 2019, urology also occurred at the top and also showed a 54% prevalence. The same was true in 2017, when urologists’ level of burnout appeared to be the highest in severity of any specialty. This tendency has been present since 2013, and urology is still the most at-risk specialty in terms of the occurrence of burnout. A study from 2014 indicated that urologists had the worst work–life balance, with only 29.3% of urologists being satisfied in this aspect [49]. Urologists comprise a different professional group, but these studies show the tendency among workers in urology departments. In the case of a lack of studies focusing on urology nurses, we may conclude that this tendency is common among all urology healthcare providers.

Urological nurses identified 98 distinct roles, with the most common tasks being patient/family education, catheterization, intermittent catheterization, medication administration, uroflow, and assisting urologists with procedures. Nurses around the world reported many roles, and although there were many common roles, nurses are taking on advanced roles, such as performing cystoscopies, circumcisions, and prostate biopsies. Unfortunately, in contrast to the USA or some European countries, Polish urology nurses cannot perform bladder ultrasound or prostate biopsies, etc. Nursing titles vary greatly around the globe. The lack of continuity in titles may lead to confusion among nurses and the public at large [50]. This fact proves that urology nursing is still developing. In addition to these practical tasks, we need to examine nurses’ job satisfaction and burnout because there is currently a lack of studies in this area focusing on urology nurses, as mentioned above. The development urology nursing and increases in nurses’ competences may not be effective if nurses are unsatisfied, depressed, and experiencing symptoms of professional burnout, which can lead to low productivity and ultimately result in their leaving the workplace.

The research was conducted in the middle of the COVID-19 pandemic, which undoubtedly had an impact on healthcare providers, including urology nurses. Even though this was not a directly examined factor in our own study, we cannot completely rule it out because respondents may not have even realized the impact of COVID-19. Nurses who provided patient care during the COVID-19 pandemic and had close contact with infected patients were often left with inadequate protections from contamination, and had a high risk of infection, burnout, fear, anxiety, and depression. The COVID-19 pandemic outbreak led to a sharp increase in admissions and hospital treatment, and consequently influenced nurses’ workload. A previous study found that each additional patient under a nurse’s care increased the nurse’s risk of burnout by 23% [51].

In the study conducted by Wojciechowska et al., it was found that 7.4% of nurses working in surgical departments experienced a high level of professional burnout, while 81.5% had an average level, and 11.1% had a low level of professional burnout [52]. In Bartosiewicz’s et al. study, nurses reported average scores on the LBQ questionnaire, with psychophysical exhaustion at 16.00 points, disappointment at 15.43 points, lack of commitment to relationships with patients at 14.98 points, and feelings of ineffectiveness at 14.97 points [53]. Another study by Uchmanowicz et al. assessed professional burnout using the Maslach Burnout Inventory, revealing that nurses scored 34.67 out of 100 points, indicating an average level of professional burnout [54]. The studies conducted by Wojciechowska, Bartosiewicz, and Uchmanowicz not only corroborated the findings of the present study but also indicated an average level of occupational burnout.

Wieder-Huszla et al. found that a high level of professional burnout occurred among nurses working in palliative and long-term care departments [3]. Similarly, Meeusen et al. demonstrated that professional burnout occurred more frequently among nurses working in intensive care and surgical departments [55]. On the other hand, Kowalczuk et al. demonstrated that nurses working in internal medicine and pediatric departments experienced a high level of psychophysical exhaustion, while nurses working in surgical departments had an average level [56]. The study conducted by Zborowska et al. revealed a lower level of professional burnout among nurses working in pediatric departments compared to nurses working in inpatient healthcare [57]. Nurses in urology departments exhibited a lower level of professional burnout compared to nurses in the studies conducted by Kowalczuk and Wieder-Huszla, who worked in palliative care, long-term care, internal medicine, and pediatric wards. The level of occupational burnout among nurses working in urology wards was similar to that of nurses in surgical wards. In contrast, Zborowska’s study presented opposite findings regarding pediatric wards. Furthermore, in Meeusen’s study, surgical nurses had a higher level of professional burnout compared to the nurses in our study.

Bartosiewcz et al. reported that factors such as marital status and place of residence or work did not impact professional burnout [53]. Similarly, in our study, the place of residence had no impact on any aspect of professional burnout.

Hallsten et al. demonstrated that education has an impact on professional burnout [58]. However, in her study, Falba showed that education has no influence on professional burnout among nurses working in surgical wards [59]. Dębska et al. revealed that the number of courses attended by nurses working in the AOS contributed to a decrease in psychophysical exhaustion and had an impact on their sense of personal achievement [60]. In our study, we did not confirm the findings of Hallsten, Dębska, and Bartosiewicz regarding education, as our study only found an influence of the number of forms of postgraduate education on disappointment. Similarly, Falba’s study also showed no impact on education. In the study conducted by Gawęda et al., a significant portion of nurses (40%) reported that their employers did not create the necessary conditions to improve their skills, and even when nurses did improve their skills, this did not guarantee promotion [61]. This lack of opportunity for advancement may contribute to the low sense of personal achievement among nurses.

Low wages are a significant source of stress among nurses and contribute to the development of professional burnout, as confirmed by our study (which demonstrated the impact of financial satisfaction on every area of the LBQ), as well as the studies conducted by Ogińska et al. [62], Kowalczuk et al. [56], and Cegła et al. [63].

The studies conducted by Flynn et al., Lu et al., and Dhaini et al. showed no correlation between the number of patients per nurse and professional burnout [64,65,66]. However, the studies conducted by Aiken et al. and Zhou et al. revealed a high patient-to-nurse ratio, which was identified as a cause of psychoemotional exhaustion [67,68]. Akman et al. [69] and Faller et al. [70] demonstrated that a lower number of patients per nurse was associated with lower levels of professional burnout. In our study, the number of patients had an impact on psychophysical exhaustion and the lack of commitment to relationships with patients. This finding was not confirmed by the studies of Flynn and Dhaini but was confirmed by the studies of Aiken, Akman, and Faller.

Jennings demonstrated a correlation between the work pattern of 12 h shifts and increasing occupational stress [71]. A study conducted among Egyptian nurses revealed that working night shifts and the number of shifts had a significant impact on the occurrence of professional burnout [72]. Other studies have shown that nurses working on shifts are more prone to experiencing professional burnout compared to those working only morning shifts [73]. In our study, the work pattern had an impact on professional effectiveness and job satisfaction, which could be a result of the high levels of stress and fatigue. Baron and Reid found that nurses working on the shift system had an increased risk of psychological stress and severe fatigue [74]. Nurses’ work has an impact on their physical and mental well-being, the quality of their services, and patients’ safety [75].

The study conducted by Dechas et al. demonstrated that nurses who perceived their health as being in a poor condition had a fivefold higher risk of professional burnout compared to nurses who perceived their health as being in a good condition. Furthermore, nurses who perceived their health as being at an average level had a 13.7 times higher risk of professional burnout than those who perceived their health as being in a good condition [76]. This study aligns with our own study, where we found that the number of nurses’ diseases had an impact on psychophysical exhaustion.

In the study conducted by Zborowska et al., nurses obtained a score of 17.13 on the SSP scale [57]. Uchmanowicz et al. reported a score of 19.76 [54], Kołtuniuk et al. reported a score of 18.5 [77], and Kalinowska et al. reported a score of 22.23 [78]. These scores indicate an average level of job satisfaction. Studies conducted among Iranian nurses reported a score of 45.5 out of 70 points [79], while Ethiopian nurses obtained a score of 67.43 out of 124 points [80], with both indicating an average level of job satisfaction. In the study by Gawęda, nurses expressed overall satisfaction with their work [61]. Our own study found that nurses had an average level of job satisfaction, which is consistent with the findings from other studies. However, a study conducted among six Brazilian hospitals reported that nurses were not satisfied with their work [81]. A study comparing job satisfaction among nurses in Norway and Poland revealed that Norwegian nurses had higher satisfaction levels than Polish nurses. Specifically, 25% of Norwegian nurses reported high job satisfaction compared to only 1% of Polish nurses, while 16% of Norwegian nurses reported low job satisfaction compared to 75% of Polish nurses [82]. In our own study, nurses working in urology departments fell somewhere in between Norwegian and Polish nurses in terms of job satisfaction.

Sowińska et al. found no correlation between job satisfaction and wages. In their research, nurses described their wages as insufficient and dissatisfactory in relation to the requirements and efforts put into their duties. Although there was no direct relationship between job satisfaction and financial satisfaction, 54% of nurses mentioned that improving their wages could increase their job satisfaction [83]. A study among Swedish nurses revealed that they were generally satisfied with their work but unsatisfied with their low wages and the lack of opportunities to improve their professional qualifications [84]. Nurses from villages identified low wages as the main reason for their willingness to seek employment in cities [85]. Gawęda et al. also confirmed the impact of low wages on job satisfaction [61]. Brazilian nurses, however, held the opposite opinion, as they expressed satisfaction with their wages [82]. Our own study demonstrated that as wages decrease, job satisfaction also decreases, which is consistent with findings from other studies, except for the Brazilian study. Sowińska’s et al. study found no correlation, but low wages were identified as a cause of dissatisfaction.

Korompeli et al. demonstrated that nurses exhibit higher satisfaction levels when they work on the shift system compared to those working only morning shifts [86]. Dall’Ora et al. showed that nurses employed in hospitals with shifts exceeding eight hours express greater dissatisfaction than those with shorter shifts [30]. However, Skorupska-Król obtained contrasting results, suggesting that working on shifts and night shifts was less stressful [2]. Our study aligns with Dall’Ora’s research but differs from the findings of Korompeli and Skorupska-Król. Kowalczuk et al. described how 12 h shifts have a significant impact on increasing stress levels, particularly during night shifts. Prolonged work under such conditions can lead to fatigue and disruptions in circadian rhythm, potentially compromising the quality of care [56]. Jennings described an increase in stress levels among nurses working on shifts [72]. This heightened stress may positively influence job satisfaction.

Faragher et al. demonstrated a strong relationship between health and job satisfaction. Mental states, such as professional burnout and low self-esteem, had a higher impact than physical health [87]. However, our study showed no correlation between the number of diseases a nurse faced and the level of job satisfaction.

Dziąbek et al. found no relationship between the place of residence and job satisfaction [88]. Conversely, Kalinowska et al. reported that nurses from rural areas exhibited a higher level of job satisfaction compared to those working in medium-sized cities [64]. Nurses in the study conducted by Dotson et al. indicated that job satisfaction was a reason for their remaining in their occupation [85]. In our study, we found no correlation between job satisfaction and the place of work or residence.

Gawęda et al. revealed that the number of patients, the nurse’s health, the number of patients per nurse, and physical and mental strain were factors contributing to decreased job satisfaction [61]. Similarly, nurses in the study by Skorupska-Król et al. identified staff shortages as the most stressful factor in their shifts [2]. However, our study showed no relationship between the number of patients and job satisfaction.

One of the limitations of this study was the SARS-CoV-2 pandemic, which resulted in limited access to hospitals and nurses. Additionally, the lack of previous studies focusing specifically on urology departments and the absence of previous analyses including selected factors, such as the place of residence, postgraduate education, and the number of nurses’ diseases, posed certain difficulties and limitations when conducting this research.

A future line of study is to conduct research assessing job satisfaction and professional burnout after the COVID-19 pandemic on a larger sample of urology nurses and compare the results to this study. Additionally, we aim to examine the changes that occurred in nursing care for urological patients after the pandemic. Furthermore, we would like to include a comparison of the competences and tasks performed by urological nurses in Poland and their opinions as to whether they want to extend their roles to include certain activities currently performed by doctors, such as cystoscopies and prostate biopsies, as is the case in the USA.

## 5. Conclusions

The level of job satisfaction and professional burnout among nurses employed in urology departments were comparable to those observed in other departments and countries.

Financial satisfaction emerged as a significant factor influencing both job satisfaction and professional burnout, indicating the necessity for adjustments in nurses’ salaries. It also underscored that nurses do not feel adequately financially rewarded for their contributions.

The number of patients under nurses’ care had repercussions regarding psychophysical exhaustion and the lack of commitment to relationships with patients, signaling an imbalance between patient load and nursing staff. This imbalance may lead to adverse outcomes, such as the rationing of nursing care, diminished care standards, and an elevated risk of medical errors.

Healthcare institutions should prioritize factors influencing job satisfaction and the risk of professional burnout when formulating employment conditions. Their primary objectives should encompass establishing a supportive work environment and cultivating cohesive teams that are motivated to achieve organizational objectives.

## Figures and Tables

**Table 1:** The sociodemographic characteristics of the study group.

Marital Status	Frequency	Percent
Married	75	58.10%
Single	45	34.90%
Divorced	9	7.00%
Place of residence		
City	100	76.90%
Village	30	23.10%
Education		
Medium	15	11.50%
Higher undergraduate	75	57.70%
Graduate and above	40	30.80%
Work pattern		
Single shift	15	11.50%
Shift	115	88.50%
Satisfaction with the financial situation		
No	33	25.40%
Average	62	47.70%
Yes	35	26.90%
Staying on sick leave		
No	84	64.60%
Yes	46	35.40%
I want to change my job		
No	35	26.90%
Maybe	55	42.30%
Yes	40	30.80%

Source: own study.

**Table 2:** The descriptive statistics of job satisfaction and professional burnout.

LBQ	M	SD	Min	Max	Me
Psychophysical exhaustion	22.29	6.01	7.00	36.00	22.00
Lack of commitment to relationships with patients	20.02	4.93	8.00	35.00	20.00
Feeling of ineffectiveness	17.37	4.49	7.00	27.00	18.00
Disappointment	19.66	6.08	6.00	36.00	21.00
Scale of Job Satisfaction (SSP)	17.23	6.25	5.00	35.00	17.00

M—medium; SD—standard deviation; Min—minimum; Max—maximum; Me—median. Source: own study.

**Table 3:** The correlation analysis of independent variables (predictors).

	Age	Postgraduate	Work Places	Seniority	Illnesses	Work Pattern	Financial Situation	Willingness to Change
Postgraduate	0.326 ***	—						
Workplaces	0.029	0.148	—					
Work experience in the profession	0.895 ***	0.409 ***	0.015	—				
Internship in the urology department	0.745 ***	0.398 ***	−0.061	0.822 ***	-			
Number of sick	0.184 *	0.124	0.280 **	0.159				
Number of diseases	0.021	0.010	0.094	0.100	—			
Education	−0.013	0.231 **	0.093	−0.045	−0.014			
Work pattern	−0.155	−0.023	0.037	−0.152	0.067	—		
Financial satisfaction	0.054	0.043	−0.136	0.049	−0.150	0.008	—	
Staying on sick leave	0.098	−0.085	−0.051	0.116	0.314 ***	−0.085	−0.105	
Willingness to change	−0.243 **	−0.007	0.079	−0.165	0.263 **	0.175 *	−0.311 ***	—
Marital status	−0.452 ***	−0.186 *	0.174 *	−0.370 ***	0.034	0.103	−0.018	0.201 *
Place of residence	−0.084	−0.103	−0.001	−0.139	−0.023	0.084	0.139	−0.126

Note: * *p* < 0.05, ** *p* < 0.01, *** *p* < 0.001. Source: own study.

**Table 4:** The impact of sociodemographic characteristics on job satisfaction and professional burnout—analysis of variance.

Model		*SS*	*Df*	*MS*	*F*	*p*
LBQ (Psychophysical exhaustion)	Regression	1076.270	6	179.378	6.170	<0.001
	Rest	3576.199	123	29.075		
	Total	4652.469	129			
LBQ (Lack of commitment to relationships with patients)	Regression	614.506	6	102.418	5.006	<0.001
	Rest	2516.425	123	20.459		
	Total	3130.931	129			
LBQ (Feelings of ineffectiveness)	Regression	322.810	6	53.802	2.903	0.011
	Rest	2279.467	123	18.532		
	Total	2602.277	129			
LBQ (Disappointment)	Regression	997.478	6	166.246	5.413	<0.001
	Rest	3777.629	123	30.712		
	Total	4775.108	129			
SSP (Scale of Job Satisfaction)	Regression	1315.369	6	219.228	7.257	<0.001
	Rest	3715.708	123	30.209		
	Total	5031.077	129			

Note: *SS*—sum of squares; *Df*—degrees of freedom; *MS*—mean square; *F*—test statistics; *p*—statistical significance; LBQ—Link Burnout Questionnaire. Source: own study.

**Table 5:** The impact of sociodemographic characteristics on job satisfaction and professional burnout—regression analysis.

Model		*B*	*SE*	*β*	*t*	*p*
LBQ (Psychophysical exhaustion)	(Constant)	19.435	3.548		5.478	<0.001
	Place of residence	−0.613	1.153	−0.043	−0.532	0.596
	Number of forms of postgraduate education	−1.150	0.591	−0.157	−1.946	0.054
	Work system	1.552	1.496	0.083	1.037	0.302
	Number of sick	0.234	0.107	0.180	2.196	0.030 *
	Satisfaction with financial situation	−1.745	0.678	−0.211	−2.575	0.011 **
	Number of diseases	0.657	0.198	0.269	3.315	0.001 **
LBQ (Lack of commitment to relationships with patients)	(Constant)	20.133	2.976		6.765	<0.001
	Place of residence	1.036	0.967	0.089	1.072	0.286
	Number of forms of postgraduate education	−0.795	0.496	−0.132	−1.604	0.111
	Work system	0.438	1.255	0.029	0.349	0.728
	Number of sick	0.210	0.089	0.197	2.353	0.020 *
	Satisfaction with financial situation	−2.170	0.568	−0.320	−3.818	<0.001 ***
	Number of diseases	0.170	0.166	0.085	1.023	0.309
LBQ (Feeling of ineffectiveness)	(Constant)	13.553	2.833		4.785	<0.001
	Place of residence	0.713	0.921	0.067	0.775	0.440
	Number of forms of postgraduate education	−0.649	0.472	−0.118	−1.375	0.172
	Work system	2.838	1.195	0.203	2.375	0.019 *
	Number of sick	0.078	0.085	0.080	0.914	0.362
	Satisfaction with financial situation	−1.372	0.541	−0.222	−2.536	0.012 *
	Number of diseases	0.018	0.158	0.010	0.113	0.309
LBQ (Disappointment)	(Constant)	18.918	3.646		5.188	<0.001
	Place of residence	−0.958	1.185	−0.067	−0.809	0.420
	Number of forms of postgraduate education	−1.405	0.607	−0.189	−2.314	0.022 *
	Work system	1.342	1.538	0.071	0.873	0.384
	Number of sick	0.205	0.110	0.156	1.871	0.064
	Satisfaction with financial situation	−1.902	0.696	−0.227	−2.731	0.007 **
	Number of diseases	0.537	0.204	0.217	2.634	0.010 **
SSP (Scale of Job Satisfaction)	(Constant)	17.588	3.616		4.863	<0.001
	Place of residence	1.639	1.175	0.111	1.394	0.166
	Number of forms of postgraduate education	0.820	0.602	0.107	1.361	0.176
	Work system	−3.106	1.525	−0.160	−2.036	0.044 *
	Number of sick	−0.190	0.109	−0.141	−1.747	0.083
	Satisfaction with financial situation	3.025	0.691	0.352	4.380	<0.001 ***
	Number of diseases	−0.328	0.202	−0.129	−1.624	0.107

Note: *B*—non-standardized coefficient; *SE*—standard error; *β*—standardized coefficient; *t*—test statistics; *p*—statistical significance; LBQ—Link Burnout Questionnaire; SSP—Scale of Job Satisfaction. * *p* < 0.05; ** *p* < 0.01; *** *p* < 0.001. Source: own study.

**Table 6:** The impact of sociodemographic data on job satisfaction and professional burnout—matching model.

Model	*R* ^2^	Corrected *R*^2^	The Standard Error of the Estimate
LBQ (Psychophysical exhaustion)	0.231	0.194	5.39210
LBQ (Lack of commitment to relationships with patients)	0.196	0.157	4.52313
LBQ (Feelings of ineffectiveness)	0.124	0.081	4.30491
LBQ (Disappointment)	0.209	0.170	5.54188
SSP (Scale of Job Satisfaction)	0.261	0.225	5.49627

Note: *R*^2^—model fit factor; source: own study.

## Data Availability

The datasets generated during and/or analyzed during the current study are available from the corresponding author on reasonable request.

## Public Involvement Statement

No public involvement in any aspect of this research.

## Guidelines and Standards Statement

This manuscript was drafted against STOBE for cross-sectional research.
